# Benchmarking infrastructure for mutation text mining

**DOI:** 10.1186/2041-1480-5-11

**Published:** 2014-02-25

**Authors:** Artjom Klein, Alexandre Riazanov, Matthew M Hindle, Christopher JO Baker

**Affiliations:** 1 Computer Science And Applied Statistics Department, University of New Brunswick, Saint John, Canada; 2 IPSNP Computing Inc, Saint John, New Brunswick, Canada; 3 Synthetic and Systems Biology, Edinburgh University, Edinburgh, UK

## Abstract

**Background:**

Experimental research on the automatic extraction of information about mutations from texts is greatly hindered by the lack of consensus evaluation infrastructure for the testing and benchmarking of mutation text mining systems.

**Results:**

We propose a community-oriented annotation and benchmarking infrastructure to support development, testing, benchmarking, and comparison of mutation text mining systems. The design is based on semantic standards, where RDF is used to represent annotations, an OWL ontology provides an extensible schema for the data and SPARQL is used to compute various performance metrics, so that in many cases no programming is needed to analyze results from a text mining system. While large benchmark corpora for biological entity and relation extraction are focused mostly on genes, proteins, diseases, and species, our benchmarking infrastructure fills the gap for mutation information. The core infrastructure comprises (1) an ontology for modelling annotations, (2) SPARQL queries for computing performance metrics, and (3) a sizeable collection of manually curated documents, that can support mutation grounding and mutation impact extraction experiments.

**Conclusion:**

We have developed the principal infrastructure for the benchmarking of mutation text mining tasks. The use of RDF and OWL as the representation for corpora ensures extensibility. The infrastructure is suitable for out-of-the-box use in several important scenarios and is ready, in its current state, for initial community adoption.

## Introduction

### Mutation text mining

The use of knowledge derived from text mining for mentions of mutations and their consequences is increasingly important for systems biology, genomics and genotype-phenotype studies. Mutation text mining facilitates a wide range of activities in multiple scenarios including the expansion of disease-mutation database annotations [1], the development of tools predicting the impacts of mutations [2,3], the modelling of cell signalling pathways [4] and protein structure annotation [5,6]. The types of useful text mining tasks specific to mutations range from the relatively simple identification of mutation mentions [7,8], to very complex tasks such as linking (*“grounding”*) identified mutations to the corresponding genes and proteins [9-11], interpretation of the consequences of mutations in proteins [12], or identifying mutation impacts [13,14] and related phenotypes [15].

Although the demand for mutation text mining software has lead to a significant growth of the experimental research in this area, the development of such systems and the publication of results is greatly hindered by the lack of adequate benchmarking facilities. For example, in developing a mutation grounding system [11] showing an encouraging level of performance accuracy, 0.73, on a homogeneous corpus of 76 documents, the authors achieved only 0.13 on a heterogeneous corpus of larger size. When the system was reimplemented (see [16]), the authors encountered another challenge – the evaluation of the new system by comparing it to the state-of-the-art was practically unaffordable, despite the existence of similar systems, due to the lack of consensus benchmarking infrastructure.

Such challenges and evaluation issues are not unique or specific for mutation text mining, and are also present in other domains of biomedical text mining. In the following subsection, we discuss benchmarking and evaluation difficulties in biological text mining in general, which are also relevant to mutation text mining.

### Benchmarking and evaluation challenges in biomedical text mining

Benchmarks, in the form of annotated corpora and related software utilities, are usually designed and created for specific text mining tasks and support fixed, usually hard-coded, evaluation metrics. Besides quantitative and qualitative characteristics – number of entities annotated, distribution of annotation types, etc. – a corpus is characterized by the format, annotation schema (semantics of annotations, annotation types), and evaluation metrics to calculate the performance of the text mining systems. For example, the benchmark for MutationFinder [7] (one of the most popular single point mutation extractors) is in a custom tabular format, stores annotations and raw text separately, has annotations of single point mutation mentions without references to their position in text and provides three different performance metrics.

*Comparative performance evaluations* between systems and evaluation of these systems on different *gold standard data* are essential for text mining systems to be formally verified and adopted. Developers need to be able to convincingly evaluate their system’s performance by comparing their results with extensive gold-standard data-sets and results of other systems. Since systems are often integrated as sub-programs in larger text mining pipelines, third-party developers also need to make comparisons when looking for a better candidate to be integrated in their new system. Biocurators use text mining tools to pre-annotate documents for manual annotation. They also evaluate third-party tools in order to find a system which performs better on representative benchmarks.

There are several state of art challenges related specifically to comparative evaluation: 

(1) **Availability** Typically developers only publish their results but not their corpora and systems. The resources can not be re-used nor tested, because they are just not available.

(2) **Reproducibility** It is often the case that developers do not provide instructions on how to reproduce their results, or if they do, the instructions require considerable effort from the user (download and compile code, download corpus, train system on corpus, run test and evaluations). This presents a practical challenge for a person who wants to perform a comparative analysis, but does not have the specific skills or knowledge of the required tools.

(3) **Interoperability** Evaluation is hindered by the diversity and heterogeneity of formats and annotation schemas of corpora and systems. In order to compare systems, developers have to convert corpora and the systems output to appropriate formats. Definitions and implementations of evaluation metrics are often format and schema dependent. Using a different schema or modifying a schema requires re-implementation of metric calculation scripts. Re-usability is also hindered by the complexity of native corpora formats. Some of them are so idiosyncratic that special programs have to be developed to convert them to a unified format or the format used by the system being tested.

(4) **Comparability** Corpora vary in qualitative and quantitative characteristics which might significantly affect the performance evaluation results. Ideally, a text mining system must be tested on *large* corpora with *representative characteristics*.

(5) **Diversity of metrics** Text mining systems are usually evaluated by using such performance metrics as *precision* and *recall* and different flavours of these statistics are used by different system developers. For example, text mining results sometimes need to be evaluated with different granularities, e.g., the mutant protein property change may be evaluated by considering binary outcomes (*has effect* vs *no effect*) or with higher granularity when the outcome may also identify the direction of the effect – e.g., *positive effect* or *negative effect*.

### Our research goals

The lack of an adequate benchmarking infrastructure for the community is a great hindrance to the objective evaluation and, consequently, publication of mutation text mining research. Therefore, we developed an extensible and multi-purpose infrastructure, based on a consensus corpora and utilities for the community, in order to make such benchmarking and evaluation easy.

### Requirements

To orient our work, we imposed the following requirements on the infrastructure. To maximize its utility for system testing and evaluation, the infrastructure must include *a standard corpus* (a collection of manually annotated texts). As *a gold standard* we consider any manually annotated texts, with or without inter-annotator agreement analysis, which can be used for automatic training and evaluation of text mining systems. Very often small or medium-scale corpora are curated to develop text mining systems. They are not of the same quality as the community challenge standards such as BioCreative [17], CALBC [18], CRAFT [19], or GENIA [20], since they may not provide an inter-annotator agreement analysis or may not publish detailed annotation guidelines. However they can still be useful for the development of practical tools.

In order to make gold standard corpora interoperable with other corpora formats, appropriate converters must be provided. The infrastructure must also contain results of runs of different systems, in order to facilitate comparison of their performance, as well as comparative evaluation of new systems. To be useful to a larger community, the infrastructure should support *multiple mutation-related text mining tasks*, such as identifying mutations both on protein and DNA levels, mutation grounding to genes and proteins, identifying effects of mutations, etc. There should be support for annotations on different levels (to distinguish annotations on the *sentence level* where the positions of annotations in text are specified, from *document level* annotations that are assigned to the document as a whole, e.g. annotation *“in document PMID:7705350 mutation N30D is grounded to protein with UniProtID:P22643”*). Sentence level annotations are required by many important applications. For example, the curation of text-mined information about mutations intended for inclusion in databases is much more efficient if sentence level provenance is provided. However, some systems, especially early prototypes, do not provide sufficiently precise references to text fragments.

Query facilities are required to search the corpora and system results for performance analysis, data drill-down and computation of statistics, such as finding the number of annotated named entities, their types, distribution of annotation types within corpora, etc. The infrastructure must be easy to use and require only minimal effort from system developers. Ideally, many development tasks should be facilitated out-of-the-box, so that the developers do not need to create new data formats or write additional scripts in order to leverage the infrastructure.

### Article overview

In this article we report on the design and implementation of an annotation and benchmarking infrastructure to support the development, testing, benchmarking, and comparison of systems for extracting information about mutations in the text mining community. The article outline is as follows. The *Methods* Section describes our motivation for the choice of representation format, outlines the ontologies used for modelling annotations and briefly introduces our approach for calculating evaluation metrics. The *Results* Section presents details of the seed corpora, methods for the calculation of performance metrics and describes relevant utility programs. In the *Evaluation* Section we describe two case studies used to test the infrastructure. The *Future work* Section announces the forthcoming extensions to the infrastructure. Finally, the *Conclusions* Section summarizes the results and specifies how the infrastructure can be accessed.

## Related work

The needs of corpora and system availability (1) and guidelines for reproducibility (2) mentioned in the introduction strictly depend on the motivation of researchers and developers to publish their corpora, text mining systems and reproducible results. The issues of interoperability (3) and comparability (4) have been already addressed several times in the literature. In [21], six different corpora were analyzed with respect to their usage. The authors of [21] note the effect of design features and characteristics – especially the format of the corpus – on the usage rate. They empirically confirmed that corpora in more common formats are more widely used than corpora in more *ad hoc* formats. The authors of [22] also conclude that the format of a corpus is a major obstacle that hinders reuse of the corpus outside of the lab that has developed it. They write custom converters for corpus formats to demonstrate the practicability of republishing corpora for reuse.

Typically document annotations intended for testing and evaluation of text mining systems, as well as text mining results, are represented in various custom XML-based or tabular formats. In most cases there is no interoperability on the level of annotation representation (the *syntactic level*), nor is there any on the level representing annotation meanings (the *semantic level*). To overcome the problem of syntactic interoperability, some of the existing formats are supported by translators converting corpora into other popular formats (IeXML [23] or TEI [24]) which are compatible with popular annotation frameworks such as GATE [25], UIMA [26], BRAT [27], Knowtator [28].

Representing annotations in the RDF format and using OWL ontologies to model meaning of annotations is an alternative for XML and tabular formats. We know at least 2 corpora in RDF - CALBC [29] and CRAFT [19]. Moreover some text mining tools adopt RDF to model their output [13,30]. Specifically, we point out the NLP2RDF project [31] initiated to create an interchange format for linguistic tools, resources, and annotations. One of its main goals is to achieve interoperability of corpora and linguistic resources by making them available as Linked Open Data. There are also attempts to develop RDF patterns to model annotations in NLP ( [32]) and BioNLP ( [33]) communities.

The problems of interoperability, comparability and re-usability of text mining resources were specially addressed by the BioCreative group through the organization and preparation of the BioCreative Interoperability Initiative [34]. Its goals include promoting simplicity, interoperability, and broad reuse of text mining resources by introducing and popularizing a new annotation standard – *BioC*[35], an interchange format for corpora and tools in BioNLP. The authors aim to achieve minimal interoperability - using basic categories such as sentences, tokens, parts of speeches, and several named entity categories. The work is at a very early stage and currently no detailed specification of the approach is available.

The need in comparative evaluation of text mining systems was previously addressed in BioNLP community. Nine gene taggers were evaluated against five different corpora in [36]. A comparative evaluation of several protein/gene taggers was done in [37,38]. Provide a webportal and standardized infrastructure through which the evaluation of biomedical Named Entity Recognition systems can be run against different gold standard corpora. The developer of a text mining system can download, annotate and upload, for the purpose of evaluation, the gold standard corpus that best fits the text mining task under evaluation.

A practical attempt to standardize and improve the interoperability of resources in the protein-protein interaction (PPI) domain was carried out by [39]. The authors compare five PPI corpora and two PPI extraction systems and point out that the transformation of the corpora - which are in XML format and have highly idiosyncratic native schema - into the unified format was a tedious process requiring significant effort. Nevertheless, complex transformation programs were developed for the corpora, although in several cases manual disambiguation could not be avoided. One of the main findings in [39] was that methods evaluated on different corpora of different size, domains and annotations schemas vary significantly and the choice of corpus has an even larger effect on the result than the choice between different PPI extraction methods. They conclude that *“the BioNLP community faces a situation where it is difficult, if not impossible, to reliably identify the best published methods and techniques due to a lack of information on the comparability of their evaluated performance”*.

To predict and avoid the difficulties and issues identified, in particular, in the domain of PPI text mining, we propose to improve the existing situation by developing a centralised, publicly accessible multi-purpose benchmarking infrastructure for mutation text mining systems. The infrastructure comprises of (i) preliminary gold standard corpora formatted in RDF, currently including several seed corpora supporting several mutation text mining tasks, (ii) an annotation ontology modelling annotations in corpora and system results, (iii) a domain ontology modelling entities and relations between them, extracted from text, and (iv) a library of SPARQL queires for computing performance metrics.

## Methods

### Representing manual annotations and system results in RDF

XML is a standard and widely used generic format for corpora annotations and comes with a large number of tools. However, the processing of complex annotations in specific XML-based formats – parsing, storing, querying, evaluation – is usually impossible in practice with off-the-shelf XML tools without additional customization [40]. Developers of text mining systems need to create schema-specific parsers and processing scripts and change them every time the schema is changed or extended. Although syntactic interoperability is conceptually a small problem, in practical terms this is another inconvenience on the way to adoption, requiring additional work to write yet another parser for yet another format. Representing corpora and system results with different semantic annotations in RDF format makes them immediately syntactically interoperable. Thus the *syntactic interoperability* is realized by the availability of off-the-shelf RDF parsing tools and APIs.

We achieve semantic interoperability by using reference ontologies to model integrated corpora and tools. If a text mining system is compatible with the modelling documented by reference ontologies used in our framework, it is semantically interoperable with the whole benchmarking infrastructure. Our choice was additionally motivated by the fact that the RDF/OWL bundle is increasingly adopted as a medium for exchanging biomedical data. For example, it is the basis of the BIOPAX [41] format for representing biological pathway data.

These reasons make RDF a superior choice for annotation over custom XML-based formats, as the representation for our annotation, because the interoperability, reusability and extensibility of data are among the main design goals of RDF.

The advantages of using the RDF/OWL bundle include extensibility, reusability and tool availability.

#### Extensibility and reusability

Since the benchmarking infrastructure is intended for different mutation text mining tasks and all requirements can not be foreseen, the annotation representation must be extensible. Moreover, the same data may be used for different tasks (e.g., we have reused mutation impact corpora for improving mutation grounding system [16]).

The use of RDF data with classes and properties defined in OWL ontologies makes it possible to support easy integration of new corpora with annotation schemas that need not be identical, as long as they are compatible. This simply amounts to *using compatible OWL ontologies and modelling patterns for RDF*. Data defined modulo one ontology can be *simply merged* with data modulo another ontology. Moreover, additional alignments between the ontologies can be potentially provided by the annotation providers – corpus curators or text mining system developers to facilitate tighter integration of the data.

The reuse of data is in some cases also trivial because the RDF and OWL-based representation is *semantically explicit*: when a new text mining task has to be evaluated, it suffices to identify the relevant fragment of the OWL ontology.

#### Tool availability

RDF and OWL are popular open formats and supported by a large number of open source and commercial tools. Minimally the following types of tools and resources can be leveraged for the purpose of text mining annotation processing. The **SPARQL query language**[42] can be directly used for calculating system performance metrics as well as for various drill-down searches in the gold standard corpora. There is no need to implement custom query mechanisms. Multiple implementations of **RDF databases** – *triplestores* (see, e.g., [43]) – are available that facilitate efficient storing and querying of large volumes of annotations. **RDF and OWL APIs** (see, e.g., [44]) for multiple programming languages, including Java, C++, Perl and Python, facilitate easy programmatic generation and manipulation of corpus annotations or RDF data representing text mining results. **OWL reasoners** (see, e.g., [45]) can be used for data integrity checking.

The available RDF/OWL tools facilitate out-of-the-box usage of annotations and system results in the main use scenarios, such as system testing and evaluation.

### Core ontologies and modelling

The schema of our benchmarking infrastructure comprises two ontologies: (1) an annotation ontology modelling annotations in corpora and system results, and (2) a domain ontology modelling entities and their relations extracted from text. We briefly discuss the ontologies here.

#### Annotation ontology

The Annotation Ontology (AO) [33] is an open-source ontology for annotating scientific documents on the Web. We use AO to model corpora annotations as well as the relevant parts of the text mining system results. Our annotations are metadata anchored to whole documents or specific fragments of texts. They are characterized by type and optional features. In the AO, annotations are resources and realized as instances of the class Annotation. Each annotation has a hasTopic property. The value of the property is an entity extracted from text, e.g. mutation, protein, etc. This entity represents the type of the annotation.

We distinguish between two kinds of annotations: (1) *Document level annotations* are not anchored to specific fragments of text. They annotate a document as a whole, e.g. “mutation A is contained in document B”, “protein A is the topic of document B”. The annotations are linked to documents via the annotatesDocument property. (2) *Text level annotations* are anchored to specific fragments of text, e.g., “Mutation A appears in document B at position P”. Text level annotations are linked to text via instances of the TextSelector class. Text selector identifies a text fragment by its positions in the text or by its context. The property context binds annotations with text selectors.

#### Domain ontology

The Mutation Impact Extraction Ontology (MIEO) [46] is central to our infrastructure. It currently describes classes and properties necessary to represent core types of information about mutations on the protein level, identified in texts, and identified impacts of mutations on the molecular functions of proteins. For example, AminoAcidSequenceChange is the class for mutations on the protein level. Instances of ProteinVariant are most specific types of protein molecules that completely identify the corresponding amino acid sequences. Instances of ProteinPropertyChange represent identified changes of protein properties that can be linked to (1) the properties that change, (2) the corresponding documents and specific text fragments, and (3) the mutations they result from. To characterize a property change, e.g., as positive, which may correspond to increased activity, we can use the subclass PositiveProteinPropertyChange. Protein properties, such as molecular functions, are also modelled as individuals whose types are currently imported from the Gene Ontology [47].

Note that some of the mutation tasks we are interested in are related to the extraction of relations between entities rather than just identifying some entities of interest. We use custom reification for such relations, in particular to facilitate linking them to documents and more specific text fragments. For example, extracted statements of mutations impacting protein properties are represented as instances of the class StatementOfMutationEffect instead of just straightforwardly linking the involved entities with appropriate non-reified predicates.

For better interoperability, our MIEO uses the Semanticscience Integrated Ontology (SIO) [48] as an upper ontology, and the LSRN ontology [49] to represent records and identifiers from stantard Life Sciences databases, as illustrated in the next section.

#### Modelling example

**Figure 1:**
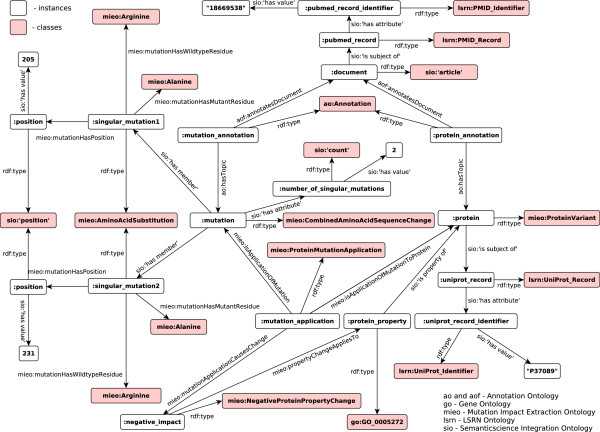
**Modelling example.** Document with PMID:18669538 reports a combined mutation (R205A and R231A) which impacts protein P37089 resulting in a negative effect on the protein property GO_0005272.

We provide an RDF graph (Figure 1) as an example of how annotated data is represented in our framework. Note that non-mnemonic ontological identifiers are replaced with pseudo-identifiers using the corresponding labels: e.g., sio:SIO_000011 and sio:SIO_000300 are replaced respectively with sio:’has attribute’ and sio:’has value’. Table 1 shows main ontologies and their namespace prefixes used in the benchmarking infrastructure.

**Table 1:** Main ontologies used in the benchmarking infrastructure for mutation text mining

**Prefix**	**URL**
ao	http://purl.org/ao/
aof	http://purl.org/ao/foaf/
aos	http://purl.org/ao/selectors/
lsrn	http://purl.oclc.org/SADI/LSRN/
mieo	http://cbakerlab.unbsj.ca:8080/ontologies/mutation-impact-extraction-ontology.owl
sio	http://semanticscience.org/resource/

Note that, for simplicity, the RDF data in this example are in “flat” RDF, i.e., they are not segmented into separate *named graphs*[50]. In practice, we would like to distinguish the gold standard data from system results, and separate results coming from different systems or different experiments. To this end, we place results from different experiments and gold standard data from different corpora in separate named graphs.

### Performance metric computation with SPARQL

An infrastructure intended for benchmarking and evaluation must support the computation of performance metrics, such as precision and recall. Different variants of these statistics vary in different systems, by their domains, level of specificity and granularity. For example, [51] proposes over 15 different metrics for evaluation of protein mutation extraction systems. Our infrastructure has to be sufficiently flexible to accommodate many different uses. This is achieved by using SPARQL to retrieve entities, such as different flavours of true and false positives, that need to be counted in order to calculate a particular metric. The current version of SPARQL (1.1) offers a sufficient degree of flexibility. In particular, the *negation-as-failure* related features – FILTER NOT EXISTS and MINUS – facilitate easy qualification of some system results as *false positives* by checking whether they are absent from the gold standard data, as will be illustrated in the *Evaluation* Section.

### Design of the seed corpora

To facilitate a preliminary evaluation of our infrastructure, we initiated it with several corpora supporting several mutation text mining tasks: (1) mutation grounding to proteins, (2) extraction of mutation impacts on molecular functions of proteins, (3) recognition of *impact sentences* – sentences which describe mutation impacts, and (4) grounding of impact sentences to mutations.

The document annotations for mutation grounding identify extracted mutations and proteins, and relations between them. The annotations for mutation impact extraction additionally identify molecular functions of proteins and changes of these properties causally linked to some mutations, and provide references to supporting text fragments. The annotated mutation impact sentences and mutations associated with them support tasks (3) and (4).

## Results

### Corpora

#### Profile of corpora

##### EnzyMiner-based corpus

One of our seed corpora was based on an extract from the EnzyMiner [52] database of publication abstracts. It was annotated manually and comprised 38 semi-randomly selected full text documents with 176 different singular mutations linked to 48 different protein sequences. The selection was adjusted to ensure maximal diversity by having documents with proteins from all enzyme families and 24 different species. The corpus contained 440 statements (occurrences of impact information in text), 57 molecular functions and 20 combined mutations. We will refer to it as “the EnzyMiner corpus”.

The documents were annotated with mutation impact information which includes: 

1. Identified **protein-level mutations**, in the form of singular amino acid substitutions. They are represented as triples specifying the wild type and mutant residues, and the absolute positions of the mutations on the corresponding amino acid sequences. For situations when the aggregate effects of several simultaneous amino acid substitutions on a protein are reported in the document, we allowed them to be expressed as *combined mutations*, which are conceptually just sets of singular amino acid substitutions and are analogous to *mutation series* in [51]. They must not be confused with a collections of single-point mutations on the same protein, whose effects on the protein are considered separately.

2. **Proteins** to which the mutations are related, identified with UniProt IDs. The host organisms and sets of specific protein sequences can be identified via the UniProt IDs.

3. **Protein properties** specified as Gene Ontology molecular function classes.

4. **Mutation impacts** on molecular functions of proteins are qualified as *positive*, *negative* or *neutral*.

5. **Text fragments** from which the information was extracted. Typical fragments contain mentions of protein properties, impact directionality words, such as “increased” or “worse”, mutation mentions, protein and organism names, etc.

6. **Documents** identified with PubMed identifiers.

The corpus was used in [16] to improve the mutation grounding algorithm in a mutation impact extraction system.

##### DHLA corpus

This is a small corpus comprising 13 documents with 52 unique (per document) mutations on Haloalkane Dehalogenases, manually annotated similarly to the EnzyMiner documents. Unlike Enzyminer, DHLA corpus does not provide annotations on text. The corpus was used to develop a mutation grounding prototype described in [11]. It mentions proteins from four different organisms and has four different UniProt IDs in the annotations.

##### COSMIC-based corpus

We have an extract from the COSMIC database [53] containing 63 documents for three target genes: FGFR3, MEN1 and PIK3CA. Unlike the EnzyMiner and the DHLA corpora, this corpus does not identify mutation impacts, although it links mutations to proteins and, thus, is suitable for mutation grounding benchmarking. The corpus was used to test mutation-grounding prototype described in [11]. We point out that the PIK3CA, FGFR3 and MEN1 corpora were developed for the study of mutations specifically on these genes in humans, and therefore unique UniProt IDs are specified in each of the three annotation subsets.

##### KinMutBase-based corpus

We retrieved 201 documents annotated with singular amino acid substitutions grounded to proteins, from the KinMutBase [54] database. We additionally curated the selection by running MutationFinder [7], which is a reliable tool for this purpose due to its very high recall, and comparing the results with the annotations in the database. Based on this comparison, we discarded about 70 documents that appear annotated with protein-level mutations that are not mentioned directly and are likely to have been inferred from SNPs by the curators. The final size of the corpus is 128 documents. In total, we have 271 mutations linked to 26 different UniProt identifiers. The corpus was used in [16] to test mutation-grounding system.

##### The Impact sub-corpus of the Open Mutation Miner corpus

This corpus, OMM Impact, containing 40 documents was used in [13] to test the Open Mutation Miner system. It contains impact sentence annotations with the EC codes of enzymes, host organisms and mutations. An impact sentence describes a mutation impact on a protein property and does not necessarily contain a mutation mention. 48 of 2045 impact sentences were not grounded to mutations. If a sentence contained several impact mentions, it was annotated several times. Unlike the Enzyminer corpus, the OMM Impact corpus was not annotated with protein properties or mutation impact direction.

#### Corpora statistics

The statistics for the corpora are summarized in Tables 2 and 3.

**Table 2:** Corpus statistics for the mutation grounding task

	**Number of documents**	**UniProt IDs**	**Mutations**^∗^
EnzyMiner	38	49	176
KinMutBase	128	26	271
DHLA	13	4	52
PIK3CA	30	1	169
FGFR3	26	1	174
MEN1	7	1	22

(^∗^) - unique per document.

**Table 3:** Corpus statistics for mutation impact extraction tasks

	**Number of**	**Impact**	**Impacts**^∗^**(mutation,**	**Impact**	**Impact sentences**
	**documents**	**mutations**^∗^	**protein property,**	**sentences**	**grounded to mutations**
			**impact direction)**		
OMM Impact	40	223	-	2045	1997
EnzyMiner	38	172	282	440	440
DHLA	13	52^∗∗^	73	-	-

(^∗^) - Unique per document.

(^∗∗^) - The OMM Impact and Enzyminer corpora contain single point mutations as well as combined mutations. There are only single point mutations in the DHLA corpus.

Most of the seed corpora do not provide inter-annotator agreement analysis or do not publish detailed annotation guidelines. In this context we define them as a *preliminary* gold standard corpora because their quality is likely to be improved in further revisions.

All the corpora were developed in the context of other projects and were presented in different tabular formats. We converted them to RDF according to the modelling adopted by the benchmarking infrastructure. There were many overlaps in their annotation schemas (see Table 4), which allowed for straightforward conversion.

**Table 4:** Corpus schemas

	**Schema (Table columns)**
EnzyMiner	PMID, UniProt ID, mutation, protein property,
	impact direction, impact sentence
DHLA	PMID, UniProt ID, mutation, protein property,
	impact direction
KinMutBase	PMID, UniProt ID, mutation
PIK3CA	PMID, UniProt ID, mutation
FGFR3	PMID, UniProt ID, mutation
MEN1	PMID, UniProt ID, mutation
OMM Impact	PMID, EC number, mutation, impact sentence

The mutation-protein grounding annotations in all seed corpora were represented as triples <document id, mutation id, protein id > and were annotated on the document level, i.e. no in-text annotations. All the mutations were protein-level single point mutations and were normalized to the amino acid sequence change format [55] recommended by the Human Genome Variation Society (HGVS) [56]. An example of this notation is “N30A”. In general, the first letter is the one-letter code of the wild type amino acid, the number is the position of the amino acid from the beginning of protein sequence, and the second letter is the one-letter code of the amino acid present in the mutation. Proteins were normalized to UniProt identifiers (except OMM Impact corpus in which mutations are grounded to enzymes normalized to EC identifiers [57]).

We note that the majority of annotations were document-level annotations and all entities were normalized. In many cases, the semantic modelling of annotations was straightforward. We discuss different several semantic interoperability issues that we faced. First, there are several inconsistencies in annotations: (1) The OMM Impact corpus provides impact sentence annotations and, in contrast to the Enzyminer corpus, it does not distinguish between different types of impacts or protein properties. (2) The DHLA corpus does not annotate text sentences with impact facts, but represents them on the document level (see Table 3). (3) Not all mutations in the Enzyminer corpus are grounded to UniProt identifiers. The corpora also differs in their text fragment boundaries. For example, in the OMM Impact corpus sentences with multiple impact mentions are annotated multiple times, whereas in the Enzyminer corpus only relevant sentence fragments are annotated. Another issue we faced was resolution to different identifiers, e.g proteins are normalized to EC numbers in the OMM Impact corpus and to UniProt IDs in other corpora. Moreover, we observed some inconsistency in the annotations of categories of protein properties. For example, the Enzyminer corpus is annotated with protein molecular functions (normalized to Gene Ontology classes) and a couple of kinetic properties such as the catalytic rate constant (Kcat) and the Michaelis constant (Km). In contrast, the OMM Impact corpus is annotated with sentences containing more protein properties, such as the maximal speed of activity (Vmax), dissociation constant (Kd) and thermostability.

These semantic interoperability issues were resolved by modelling (e.g., to model impact text fragments we used the more general class *String* instead of the class *Sentence*; inconsistent information was omitted), and by designing customized benchmarking SPARQL queries (e.g., we selected only grounded mutations - where UniProt IDs are present - in the Enzyminer corpus when evaluating mutation grounding task).

Although there were no ambiguous cases in the seed corpora, RDF naturally supports polysemous annotations. For example, if different UniProt identifiers are assigned to a protein by a curator, additional triples can be simply added to the protein node. We are not imposing any constraint to avoid ambiguous representation because new source corpora may support ambiguity. Although SPARQL can handle ambiguity relatively easily, this must be considered when writing SPARQL queries.

### RDF database

The RDF files representing our corpora are already relatively large, so for the purposes of efficient SPARQL querying we deployed the data to a Sesame triplestore [43]. Users have the option of downloading the RDF data and using their own querying machinery, or accessing our DB via a public SPARQL endpoint. The details can be found on the project portal [58].

### SPARQL queries for performance metrics

To implement and illustrate the idea of using SPARQL for performance metric computation, we formulated several SPARQL queries sufficient for computing precision and recall for systems implementing four text mining tasks: 

**(T1) Mutation grounding to proteins.** The results are mutation and protein pairs with the corresponding UniProt IDs that identify protein sequences. We adopted the definitions of precision and recall for this task from [14]: *precision* was defined as the number of correctly grounded mutations over all grounded mutations and *recall* was defined as the number of correctly grounded mutations over all uniquely mentioned mutations.

**(T2) Extraction of mutation impacts on molecular functions of proteins –*mutation-impact relations*.** The metrics were also taken from [14]. For mutation-impact relations, *precision* was defined as the number of correct relations over all retrieved relations and *recall* was defined as the number of correct relations over all uniquely mentioned relations. In order for an extracted mutation-impact relation to be considered correct all the parts have to be correct, i.e., the affected protein property, the direction of the impact and the cause mutation. If the protein property was a molecular function, it had to be normalised by grounding to Gene Ontology.

**(T3) Impact sentence recognition.** This task was evaluated in [13]. *Precision* was defined as the number of correctly identified impact sentences over all recognized impact sentences. The sentence is correctly identified if it matches the manual annotation.

**(T4) Grounding impact sentences to mutations.** This task was also considered in [13]. *Accuracy* for this task was defined as the fraction of correctly identified impact sentences, grounded to correct mutations, over all correctly identified impact sentences.

For each task we wrote (1) a SPARQL query that selects relevant annotations in the gold standard data, representing correct cases, (2) a SPARQL query that selectes all relevant/retrieved results of the text mining system being evaluated, and (3) a SPARQL query that selectes only correct results. These selections were enough to calculate precision and recall. A slightly simplified version of the query used to select the correct results from mutation-impact relation extraction can be found in the Additional file 1. Full versions of the implemented queries for performance metrics are available from the project Web page [58].

### Utilities

As a part of our infrastructure, we created a small set of simple utilities facilitating access to the data. The *Sesame loader* and *query client* are simple command line applications that allow loading RDF graphs into a Sesame triplestore and executing queries from files.

The *provenance enhancement* utility helps in situations when the sources of annotation data only provide fragments of texts as provenance, without specifying their positions in the text, such as in the Enzyminer and OMM Impact corpora. Note that the annotations in the OMM Impact corpus have position numbers, but, since the original text is not provided, the alignment of annotations with the original text still requires an additional simple program. We implemented a procedure to align corresponding text fragments based on their similarity. To calculate a similarity score for two fragment candidates we use the implementation of the Levenshtein distance algorithm from Lucene [59]. We normalized the cases of letters before applying the measure. If fragments had different lengths, we calculated the similarity score for their overlap. We avoided multiple alignments – each text fragment in a system’s results must be unambiguously aligned to the manual annotations. Two fragments get aligned if the similarity score is above the threshold. We used a threshold equal to 0.83 to boost the precision on the Enzyminer corpus used for training to 100%. The price for this is a slight decrease in recall, which still reaches 0.99. The procedure achieved >0.99 for both precision and recall on the test corpus – the OMM Impact corpus.

## Evaluation

### Case study: Improving a mutation impact extraction system

In order to test the usability and validate the utility of our infrastructure, we have applied it to the testing and iterative performance evaluation within a project dedicated to the development of a robust mutation impact extraction system [14], and to the evaluation of a mutation grounding subtask, intended for publication (see [16]). The purpose of the system is to identify protein-level mutations, ground them to the corresponding UniProt IDs and, most importantly, to identify which properties of the proteins are affected and how, when this information is present in the processed document.

Since early versions of the system already produced output in RDF, modelled according to an ontology similar to MIEO, it was straightforward to adjust the system to produce output in a format compatible with our infrastructure. This was the major prerequisite to enable the evaluation of the system on our gold standard corpora and the subsequent comparison of results from different versions of the mutation grounding system.

Although the system previously showed reasonable performance on 76 documents, the performance on the larger and more representative data set comprising the Enzyminer and KinMutBase corpora was very low. After an investigation in which we relied heavily on the analysis of system runs based on our annotations, including the provenance information, we identified the mutation grounding module as a major performance bottleneck having only 0.32 precision and 0.08 recall. We therefore focused our attention on the mutation grounding subtask, and our infrastructure was instrumental in this analysis because the task was also supported by the available manual annotations, and helped us to eventually improve the performance to 0.83 precision and 0.82 recall. More details on this effort can be found in [16].

The expressivity of SPARQL proved especially useful in this effort. An example of identifying *false-negatives* – cases presented in gold standard and absent from system results is available in Additional file 1.

### Case study: Comparative evaluation of mutation impact extraction systems

To investigate the potential of the infrastructure for comparative evaluation and analysis, we adapted the Open Mutation Miner (OMM) system [13] to produce outputs compatible with our infrastructure and compared the system with the mutation impact extraction system (MIES) [14] discussed in the previous subsection. Table 5 displays functional characteristics of both systems.

**Table 5:** Mutation impact extraction systems

	**OMM**	**MIES**
Mutation recognition	+	+
Mutation series recognition	+	-
Mutation-protein grounding	-	+
Impact sentence recognition	+	+
Impact sentence grounding to mutation	+	+
Protein property recognition and normalization	+	+
Impact direction recognition	+	+
Physical quantity recognition	+	-
Protein property-Physical quantity grounding	+	-

Previously, MIES was evaluated on the DHLA corpus (see [14] for details) using the metrics corresponding to the task T2 (*Extraction of mutation impacts on molecular functions of proteins*). The system achieved 0.86 precision and 0.34 recall. OMM was tested on the OMM Impact corpus (see [13] for details) using the metrics for T3 (*Impact sentence recognition*) and T4 (*Grounding impact sentences to mutations*). The performance of OMM was 0.71 precision and 0.714 recall on the former task and 0.77 accuracy on the latter task. We undertook a cross-evaluation of the systems – MIES on the OMM Impact corpus and OMM on the DHLA corpus. Moreover, both systems were evaluated on the new Enzyminer corpus. Technically, this was achieved by loading the corpora and system results into a Sesame triplestore and running the implemented SPARQL queries, to obtain metrics, using the Sesame Workbench web interface. The results of all the experiments are shown in Table 6.

**Table 6:** Mutation impact extraction systems: evaluation results (micro averaging)

**Task 1: Mutation Impact Extraction (P/R)**		
	**Enzyminer**	**DHLA**
OMM	0.03/0.02	0.34/0.29
MIES	0.21/0.05	0.78/0.44
**Task 2: Impact Sentence Recognition (P/R)**		
	**Enzyminer**	**OMM impact**
OMM	0.59/0.32	0.76/0.63
MIES	0.17/0.12	0.28/0.05
**Task 3: Impact Sentence Grounding (A)**		
	**Enzyminer**	**OMM impact**
OMM	0.59	0.71
MIES	0.86	0.69

P/R – precision/recall. A – accuracy.

Here we summarize our findings from the comparative evaluation for both systems. On the mutation impact extraction task both systems had low performance for Enzyminer and significantly better performance on the DHLA corpus. This can be explained by the presence of heterogeneity in the Enzyminer corpus which has 57 different protein molecular functions (compared to just one in DHLA) and the low performance of the current versions of both systems on the grounding of protein molecular functions. The grounding of molecular functions – normalization of molecular functions by assigning Gene Ontology classes to them – remains a very challenging task because the rich hierarchy of classes makes determining exact specific GO classes nontrivial.

MIES shows low results on the *Impact Sentence Recognition* task. MIES impact-extraction rules were trained on the DHLA corpus of 73 mutation-impact relations and consequently failed on a corpus several times larger. OMM was trained on a larger data set and, as a result, performed relatively well on both corpora. On the *Impact Sentence Grounding* task the systems performed similarly on the OMM Impact corpus (MIES - 0.69, OMM - 0.71) and MIES performed better on Enzyminer corpus (0.86 vs. 0.59).

## Future work

Our current work is focused on defining the procedures for the submission of third-party human-curated annotations and system results.

In the future, we will further stress-test the infrastructure with text mining tasks other than mutation grounding and mutation impact extraction, and more third-party mutation text mining systems. We will continue extending the ontology based on the new requirements identified through community involvement and our own research. In the near future, we also plan to extend the infrastructure to include protein properties other than molecular functions, such as enzyme kinetics, and DNA-level mutations (SNPs). New corpora for mutation mention recognition – the OMM Mutation [13], MutationFinder [60], and tmVar [8] corpora – will be integrated.

Currently the infrastructure lacks graphical representation of annotations. RDF is not easy to read by a human, so we will implement an interface to load annotated data into one of the following graphical annotation toolkits – GATE [25], UIMA [26], or BRAT [27]. This will enable visualization, browsing, manual modification and analysis of annotations. We will also consider leveraging the DOMEO tool [61] which is, to our best knowledge, the only graphical annotation toolkit supporting RDF. It represents annotations using the Annotation Ontology RDF model and is thus compatible with our benchmarking infrastructure. In order to promote our corpora, we will attempt to write converters to the most popular formats including BioC [35], GENIA XML [20], UIMA XMI [26], and IeXML [23]. The Mutation Impact Extraction Ontology will be also presented in the more common OBO [62] format.

## Conclusions

We have reported our results on the development of a community-oriented benchmarking infrastructure intended to relieve the developers of mutation text mining software from the burden of developing *ad hoc* corpora and scripts for testing, benchmarking and evaluation of multiple mutation-related text mining tasks. While other benchmark corpora for biological entity and relation extraction are focused mostly on genes, proteins, diseases and species, our benchmarking infrastructure fills the gap for mutation information. We have seeded the infrastructure with sizeable manually annotated corpora (282 documents in total). To maximize the reusability and extensibility of our infrastructure, we use RDF and OWL for the representation of annotation data and SPARQL queries as a means for flexible analysis of text mining results. The infrastructure was tested for benchmarking and comparative evaluation of mutation-impact extraction systems. We emphasize that for performance evaluation, corpora statistics calculation and analysis of results we did not need to write any programming code and have only used an off-the-shelf SPARQL engine. We have undertaken this work with the goal of *initiating a community effort*. The future evolution of the benchmarking infrastructure will be based on feedback and contributions from the community.

## Availability

The benchmark corpora, the ontologies, example outputs of our mutation text mining system, benchmarking SPARQL query templates and maintenance tools are available from the project Web page [58].

## Competing interests

The authors declare that they have no competing interests.

## Authors’ contributions

MMH created the Enzyminer corpus. AR created the first version of the Mutation Impact Extraction Ontology, concieved the idea of using SPARQL for metrics computation and drafted the initial set of SPARQL queries. AK created all other components of the infrastructure and final versions of the SPARQL queries, and integrated them. AK also conducted all the experiments and wrote the first draft of this article. CB coordinated the project and provided subject matter expertise. All authors read and approved the final manuscript.

## Supplementary Material

Additional file 1 **SPARQL query examples.** Additional file 1 contains example SPARQL queries to: (1) select the correct results from mutation-impact relation extraction, and (2) identify false negatives – cases presented in gold standard and absent from system results in evaluating mutation grounding task. Click here for file
