# Supplementary material for: Benchmarking infrastructure for mutation text mining
Source: J Biomed Semantics. 2014 Feb 25;5:11. doi: 10.1186/2041-1480-5-11 (PMC3939821; doi:10.1186/2041-1480-5-11)
Supplement: Additional file 1 — SPARQL query examples. Additional file 1 contains example SPARQL queries to: (1) select the correct results from mutation-impact relation extraction, and (2) identify false negatives – cases presented in gold standard and absent from system results in evaluating mutation grounding task. [file 2041-1480-5-11-S1.pdf]

Additional file 1

Publication: "Benchmarking infrastructure for mutation text mining"

Authors: Artjom Klein, Alexandre Riazanov, Matthew M Hindle and Christopher JO Baker

1. Example evaluation queries

We illustrate a specific example of a metric SPARQL query by presenting a slightly simplified version of the query used to select the correct results from mutation-impact relation extraction.

According to the definitions in evaluation task (T2) – *evaluation of extraction of mutation impacts on molecular functions of proteins*, a result was defined as a tuple – a *document*, a *mutation*, a *protein property* changed by the mutation, and a *direction* of the property change. If the gold standard data RDF graph contained a corresponding subgraph, the result was considered *correct*. Technically we had to compare two named RDF graphs and obtain the corresponding intersection. The resulting query below assumes that the gold-standard data is kept in the named graph `http://example.com/gold-standard.rdf` and the system results came from another named graph `http://example.com/experiment.rdf`.

Note that, as in the modelling example, we replace non-mnemonic SIO identifiers with their labels, for better readability,

```
SELECT DISTINCT ?pubmed_id ?mut_id ?protein_property_class ?property_change_class
WHERE {
    GRAPH <http://example.com/gold-standard.rdf> {
        ?document a sio:'article';
            sio:'is subject of'
                [ a lsrn:PMID_Record;
                  sio:'has attribute'
                      [ a lsrn:PMID_Identifier;
                        sio:'has value' ?pubmed_id ] . ] .

        ?ann_mutation a ao:Annotation;
            aof:annotatesDocument ?document;
            ao:hasTopic ?mutation .
        ?mutation a mieo:CombinedAminoAcidSequenceChange;
            sio:'has unique identifier'
                [ a mieo:CombinedAminoAcidSequenceChange_Identifier;
                  sio:'has value' ?mut_id ] .

        ?ann_mutation_application a ao:Annotation;
            aof:annotatesDocument ?document;
            ao:hasTopic
                [ a mieo:ProteinMutationApplication;
                  mieo:isApplicationOfMutation ?mutation ] .

        ?ann_statement_of_mutation_effect a ao:Annotation;
            aof:annotatesDocument ?document;
            ao:hasTopic
                [ a mieo:StatementOfMutationEffect;
                  mieo:arg1 ?mutation_application;
                  mieo:arg2 ?property_change ] .

        ?ann_property_change a ao:Annotation;
            aof:annotatesDocument ?document;
            ao:hasTopic ?property_change .
        ?property_change a ?property_change_class;
            mieo:propertyChangeAppliesTo ?protein_property .
```

```
        ?property_change_class rdfs:subClassOf mieo:ProteinPropertyChange .

        ?ann_protein_property a ao:Annotation;
            aof:annotatesDocument ?document;
            ao:hasTopic ?protein_property .
        ?protein_property a ?protein_property_class .
        ?protein_property_class rdfs:subClassOf mieo:ProteinProperty .
    }
    GRAPH <http://example.com/experiment.rdf> {
        ?document2 a sio:'article';
            sio:'is subject of'
                [ a lsrn:PMID_Record;
                  sio:'has attribute'
                      [ a lsrn:PMID_Identifier;
                        sio:'has value' ?pubmed_id ] . ] .

        ?ann_mutation2 a ao:Annotation;
            aof:annotatesDocument ?document2;
            ao:hasTopic ?mutation2 .
        ?mutation2 a mieo:CombinedAminoAcidSequenceChange;
            sio:'has unique identifier'
                [ a mieo:CombinedAminoAcidSequenceChange_Identifier;
                  sio:'has value' ?mut_id ] .

        ?ann_mutation_application2 a ao:Annotation;
            aof:annotatesDocument ?document2;
            ao:hasTopic
                [ a mieo:ProteinMutationApplication;
                  mieo:isApplicationOfMutation ?mutation2 ] .


        ?ann_statement_of_mutation_effect2 a ao:Annotation;
            aof:annotatesDocument ?document2;
            ao:hasTopic
                [ a mieo:StatementOfMutationEffect;
                  mieo:arg1 ?mutation_application2;
                  mieo:arg2 ?property_change2 ] .

        ?ann_property_change2 a ao:Annotation;
            aof:annotatesDocument ?document2;
            ao:hasTopic ?property_change2 .
        ?property_change2 a ?property_change_class;
            mieo:propertyChangeAppliesTo ?protein_property2 .

        ?property_change_class rdfs:subClassOf mieo:ProteinPropertyChange .

        ?ann_protein_property2 a ao:Annotation;
            aof:annotatesDocument ?document2;
            ao:hasTopic ?protein_property2 .
        ?protein_property2 a ?protein_property_class .
        ?protein_property_class rdfs:subClassOf mieo:ProteinProperty .
    }
}
```

We comment briefly on the query composition. The two halves of the query (lines 5-35 and 36-66) correspond to the selection of relevant data from the gold standard corpora and from the experimental system results. Since our goal was to select only *correct* results, the two selections were joined on the instances of the variables `?pubmed_id` (identifying documents), `?wt_residue`, `?mut_residue` and `?position_value` (for the wild-type and mutant residues, and positions of the corresponding mutations), `?protein_property_class` (identifying studied properties) and `?property_change_class` (identifying the direction of the property change).

Note that the query could only be used to implement *micro averaging* that treats the whole corpus as one large document. If, for some reason, we were interested in *macro averaging* or needed to see performance

results for separate documents, we could have additionally grouped the results by the PubMed identifier values.

The following SPARQL query retrieved the correct results of *Impact Sentence Recognition* (T3). `?text_selector` identifies the fragment of text referring to an impact modelled as an instance of `ProteinPropertyChange` class. Since impact sentences in the available corpora did not have exact start and end positions, we implemented an alignment procedure (see *Utilities* section for details) to match corresponding text fragments and connect corresponding `text selectors` via instance of `StringSimilarity`. Alignment of similar text fragments were applied before running the query.

```
SELECT DISTINCT ?pubmed_id ?property_change ?text_selector
WHERE {
  GRAPH <http://example.com/gold-standard.rdf> {
      ?document a sio:'article';
          sio:'is subject of'
              [ a lsrn:PMID_Record;
                sio:'has attribute'
                    [ a lsrn:PMID_Identifier;
                      sio:'has value' ?pubmed_id ] . ] .

      ?ann a ao:Annotation;
          aof:annotatesDocument ?document;
          ao:hasTopic ?property_change .

      ?property_change a ?property_change_class .
      ?property_change_class rdfs:subClassOf mieo:ProteinPropertyChange .

      ?ann ao:context ?text_selector .
      ?text_selector a aos:TextSelector .
  }
  GRAPH <http://example.com/experiment.rdf> {
      ?document2 a sio:'article';
          sio:'is subject of'
              [ a lsrn:PMID_Record;
                sio:'has attribute'
                    [ a lsrn:PMID_Identifier;
                      sio:'has value' ?pubmed_id ] . ] .

      ?ann2 a ao:Annotation;
          aof:annotatesDocument ?document2;
          ao:hasTopic ?property_change2 .
      ?property_change2 a ?property_change_class2 .
      ?property_change_class2 rdfs:subClassOf mieo:ProteinPropertyChange .

      ?ann2 ao:context ?text_selector2 .
      ?text_selector2 a aos:TextSelector .
   }
   ?sim a mieo:StringSimilarity .
   ?text_selector sio:'has attribute' ?sim .
   ?text_selector2 sio:'has attribute' ?sim .
}
```

Since precision and recall formulas represent relatively simple arithmetic, they can be also calculated in a SPARQL query combining the SPARQL queries calculating correct, retrieved, and relevant result sets, if this is convenient.

2. Example analysis query

We found SPARQL useful in analysis of results and helping us in debugging tasks. E.g. the SPARQL negation-related features proved especially useful because they allowed us to identify *false negatives* – cases presented in gold standard and absent from system results, thus identifying potential targets for optimisation. The following query represents such a use case where the `FILTER NOT EXISTS` feature is applied to exclude correct system results for mutation grounding from the set of all results:

```
SELECT DISTINCT ?pubmed_id ?wt_residue ?position_value ?mut_residue ?uniprot_record_id
WHERE {
  GRAPH <http://example.com/gold-standard.rdf> {
      ?document a sio:'article';
          sio:'is subject of'
              [ a lsrn:PMID_Record;
                sio:'has attribute'
                    [ a lsrn:PMID_Identifier;
                      sio:'has value' ?pubmed_id ] . ] .

      ?ann_mutation a ao:Annotation;
          aof:annotatesDocument ?document;
          ao:hasTopic ?mutation .
      ?mutation a mieo:CombinedAminoAcidSequenceChange;
          sio:'has member' ?singular_mutation .
      ?singular_mutation a mieo:AminoAcidSubstitution;
          mieo:mutationHasWildtypeResidue ?wt_residue;
          mieo:mutationHasMutantResidue ?mut_residue;
          mieo:mutationHasPosition
               [ a sio:'position';
                 sio:'has value' ?position_value ] .

     ?ann_mutation_application a ao:Annotation;
         aof:annotatesDocument ?document;
         ao:hasTopic
             [ a mieo:ProteinMutationApplication;
               mieo:isApplicationOfMutation ?mutation;
               mieo:isApplicationOfMutationToProtein ?protein ] .

     ?ann_protein a ao:Annotation;
         aof:annotatesDocument ?document;
         ao:hasTopic ?protein .
     ?protein a mieo:ProteinVariant;
         sio:'is subject of'
             [ a lsrn:UniProt_Record;
               sio:'has attribute'
                   [ a lsrn:UniProt_Identifier;
                     sio:'has value' ?uniprot_record_id ] . ] .
     FILTER (?uniprot_record_id != "")
  }
  FILTER NOT EXISTS {
      GRAPH <http://example.com/experiment.rdf> {
          ?document2 a sio:'article';
              sio:'is subject of'
                 [ a lsrn:PMID_Record;
                   sio:'has attribute'
                       [ a lsrn:PMID_Identifier;
                         sio:'has value' ?pubmed_id ] . ] .

         ?ann_mutation2 a ao:Annotation;
             aof:annotatesDocument ?document2;
             ao:hasTopic ?mutation2 .
         ?mutation2 a mieo:CombinedAminoAcidSequenceChange;
             sio:'has member' ?singular_mutation2 .
         ?singular_mutation2 a mieo:AminoAcidSubstitution;
             mieo:mutationHasWildtypeResidue ?wt_residue;
             mieo:mutationHasMutantResidue ?mut_residue;
             mieo:mutationHasPosition
                 [ a sio:'position';
                   sio:'has value' ?position_value ] .
```

```
        ?ann_mutation_application2 a ao:Annotation;
            aof:annotatesDocument ?document2;
            ao:hasTopic
                [ a mieo:ProteinMutationApplication;
                  mieo:isApplicationOfMutation ?mutation2;
                  mieo:isApplicationOfMutationToProtein ?protein2 ] .

        ?ann_protein2 a ao:Annotation;
            aof:annotatesDocument ?document2;
            ao:hasTopic ?protein2 .
        ?protein2 a mieo:ProteinVariant;
            sio:'is subject of'
                [ a lsrn:UniProt_Record;
                  sio:'has attribute'
                      [ a lsrn:UniProt_Identifier;
                        sio:'has value' ?uniprot_record_id ] . ] .
        FILTER (?uniprot_record_id != "")
      }
  }
}
```
